# Clinical Applications of the History of Medicine in Muslim-Majority Nations

**DOI:** 10.1093/jhmas/jrac039

**Published:** 2023-01-04

**Authors:** Alan S Weber

**Affiliations:** Weill Cornell Medical College in Qatar, QA

**Keywords:** clinical practice and history of medicine, history of medicine – uses, Islamic history of medicine, history of medicine in medical education

## Abstract

Since the early twentieth century, a number of physicians and professional historians have argued for the integration of the history of medicine into both medical education and clinical practice. After the supplanting of the humoral model of medicine in favor of the germ theory of disease in the late nineteenth century, medical school administrators have repeatedly asked medical historians for their rationale for studying “outdated science” in medical training programs beyond antiquarianism and knowledge for knowledge’s sake. However, a number of arguments can be adduced for the use and relevance of the history of medicine, including the observations that history: 1) provides examples of inspiring or highly ethical individuals who can serve as role models in practitioner identity formation; 2) helps to develop critical analytical skills and other modes of humanistic thought and behavior directly relevant to patient care (e.g., empathy); 3) promotes culturally-competent care, since history informs culture; 4) encourages inquiry into the sociocultural factors that affect the development of modern medical ecosystems; 5) provides a philosophical tradition for critiquing ethics in the medical profession. This contribution specifically traces the potential uses of Islamic medical history in the clinic and medical schools in Muslim-majority countries, primarily in the Middle East.

This essay provides an overview of the value and practical applications of the history of medicine in Muslim-majority countries in medical education and clinical practice.^1^ While some of the arguments in this paper for the importance of medical history to developing well-rounded, empathetic, culturally-competent, and patient-centered healthcare practitioners have been advanced elsewhere, many of the discussions below are novel and unique due to the Islamic and Middle-Eastern cultural contexts.^2^

This contribution is based on my fifteen-year experience teaching medical history, medical ethics, medical sociology, and philosophy at an American medical college in the Middle East (Weill Cornell Medicine – Qatar) at the undergraduate and post-graduate levels. Together with colleagues, I have in addition developed and co-directed 13 ACCME-accredited workshops for clinicians on incorporating humanistic modes of thinking into clinical practice (medical history, narrative medicine, communications skills, arts in health, art and music therapy, and medical and health humanities). The ideas below are based on a literature review and my teaching modules, invited lectures, workshops, research, and courses.^3^

Although *cross-sectional* research such as interventional studies and questionnaires, as well as qualitative observational approaches borrowed from ethnography, anthropology, and sociology have proved illuminating, many aspects of the current practice of medicine and the illness experience cannot be fully understood without a *longitudinal* examination of prior history: for example, the structure of the profession and its culture, healthcare economics, health delivery systems, beliefs on the origin of disease, health behaviors, and disease models, to cite a few areas.

While research funding for the study of the history of medicine in the Middle East is limited, it is held in high esteem by both Muslim scholars and the general public; many biomedical journals in the region such as the *Saudi Medical Journal*, *Heart Views* (cardiology), and the *Aspetar Sports Journal,* devote regular columns to the history of medicine. The International Society for the History of Islamic Medicine, which sponsors an international journal and the biannual International Congress on Islamic Medical History and Ethics, was founded by Dr. Hajar Al Hajar in 2000. The Sultan Qaboos University Medical School in Oman holds an annual medical history essay contest for students each year. In addition, the immensely popular film *1001 Inventions* and later accompanying book in 2012 detailing Muslim contributions to science and technology – despite their dubious scholarly quality – have received numerous awards, and have stimulated widespread interest in the history of Islamic science and medicine among educators in the Middle East.^4^ The late Rabie E. Abdel-Halim experimented with medical history curricula in 2012 at the Alfaisal University Medical College in Riyadh. Modules included *History of Medicine During the Islamic Civilization* designed to “prove to the students how Islamic faith and teachings stimulated the minds to discover and advance scientific medical knowledge,” and *Arab Medical Poetry* “to show how poets and poet-physicians have dealt with illness, pain, disability, birth, death and dying.”^5^ Despite this obvious interest in Islamic medical history among a diverse range of scholars, practitioners, and students, the topic is not yet widely integrated into the formal medical education curricula across the Middle East.

The Tunisian historian Ibn Khaldun (1332-1406 CE) developed a rationalist and proto-scientific historiography that is compatible with modern history of medicine methodologies, and his *Muqaddimah* should be included in history of medicine curricula in medical schools. He coined and explored the concept of *˓asabiyya*, or “tribal solidarity,” which is key to understanding the paradoxical historical continuities and modern disjunctions of North African and Islamic cultures.^6^ He additionally established a rationalist strain in Islamic historiography counter to the traditional teleological, hagiographic, and theological histories of that time period. Dale’s assertion that Ibn Khaldun was “the last Greek and the first Annaliste historian” is valid in that he employed “classical logic to identify enduring socioeconomic realities underlying cultural phenomena and ephemeral events, what he describes as the ‘general conditions of regions, races and periods that constitute the historian’s foundation.’”^7^

In other words, Ibn Khaldun was working in the Greek empirical tradition of Aristotle and Hippocrates – he was searching for fundamental axioms of politics, environment, and human behavior to deduce from them general laws of historical development. Ibn Khaldun therefore serves as a valuable paradigm for humanistic and medical educators to illustrate that Islam developed an historical methodology fully compatible with, and drawing on the same intellectual traditions as, modern scientific methods. His observations additionally provide valuable insights into modern Muslim societies, much like westerners today probe Shakespeare to understand human psychology or read Max Weber to illuminate the principles of current social institutions. Excerpts from Ibn Khaldun’s works would be valuable in the syllabus of an introductory cultural competence course in Middle Eastern countries, specifically his comments on the social differences between settled and nomadic cultures, which are still being played out today (sometimes violently) in Egypt, North Africa, the Negev, Jordan, and Kuwait.

Islam has never endorsed a binary “two cultures” split in humanistic and scientific knowledge: the word *‘ilm* (knowledge, triliteral semitic root م– ل –ع, “know”) encompasses all branches of knowledge, both historical and modern, in an analogous way that the forms of the Latin root *scio, scire* (to know) denoted all areas of human knowledge before Whewell’s narrowing of this semantic field with the neologism “scientist.”^8^ However, Ibn Khaldun did distinguish between what we today call the exact or rational sciences (for example, logic and mathematics, *al-mantiq* and *al hisab*) and “al-ʿulum al-naqliyya al-wadʿiyya” or “traditionally transmitted sciences” which included theology (*kalam*), law (*fiqh*), and sayings and traditions of the Prophet (*sunnah* and *hadith*).^9^ Ibn Khaldun’s term “revealed” (al-naqliyya) clearly indicates his philosophical stance toward the continuing relevance and timeless value of divine Quranic wisdom and past human knowledge. His view that religious-based knowledge and the natural and physical sciences should be studied on an equal footing is widely accepted in theory among modern Muslim thinkers, even in the face of the dominance of engineering, medicine, and law programs in higher education in the Middle East based on western models, which is largely an artifact of colonialism.

Islamic historiography of all periods is generally not positivist and does not often subscribe to myths of progress and evolution; thus Islamic histories provide a solid foundation for valorization of past historical knowledge. Knowledge, arguments, and exempla from the past possess high status in Islamic cultures, and Muslims consciously attempt to imitate the Prophet Muhammad (SAW). Medical history formed a part of Islamic golden age science and medical writing, simply because Al-Farabi, Ibn al-Haytham, Ibn Sina, Al-Razi, and Al-Zahrawi were writing within a still living Aristotelian and Galeno-Hippocratic framework of humoralism and the commentaries on those works. The Damascus physician Ibn Abī Uṣaybiʿah wrote a historical biographical dictionary of ancient and medieval Islamic physicians in 1246 CE entitled *The Best Accounts of the Classes of Physicians*.^10^

The following factors predispose Muslims to valorize the past: 1) the Abbasid, Umayyad, and Fatimid caliphates and later Ottoman empire circa 800-1400 CE actively promoted science, technology, music, and literature, and material wealth flourished during this “Islamic Golden Age;” 2) the period of the Prophet Mohammed (SAW) and his companions is held up as an example of correct religious praxis (*sunnah*, or traditional way of life), thus the past continues to exert a considerable direct moral influence; 3) historical documents such as the Quran and the oral traditions of the *ahadith* (prophetic sayings) are viewed as eternal (thus the past and present are indistinguishable); 4) some conservative Muslims equate any aspect of modernity with westernization and colonization and pride themselves on the continuity of their traditional culture; 5) Salafist theologians aligned with national governments, for example the Al-Sheikh family in the Kingdom of Saudi Arabia (descendants of Ibn ‘Abd al-Wahhab), have co-opted the concept of *bid’ah* (innovation), which originally meant heresies (new unorthodox religious practices), to condemn any form of modern change or development in order to maintain the political status quo, thus privileging historical patterns of life. Muslims’ strong orientation to the past, in order to preserve cultural and religious continuity in the face of increasing international secularization, provides an entry way to validating the study of history and medical history as relevant topics in medical education and clinical practice.

A very simple example of how valorization and sensitivity to the past with respect to medical history can inform modern practice would be the change in philosophical speculation on the origin of life in Islamic theology in reference to abortion and the use of embryonic stem cells. The medieval legal Islamic consensus was that life began when the embryo was ensouled at 40 or 120 days and had no legal status before that time.^11^ However, modern opinions have shifted in Islamic jurisprudence, paralleling pro-life stances in Western countries such that abortion is now banned in many Muslim-majority countries except to save a woman’s life, and the use of embryos and stem cells is banned or highly restricted. Iran, however, maintains liberal policies on embryonic stem cell use and other Muslim countries are now permitting their use, undoubtedly due to potential economic and health benefits deriving from stem cell therapies.^12^ Muslim practitioners and medical students would greatly benefit from learning about the historical evolution of philosophical ideas and legal precedents concerning the origin, purpose, and status of life in Islam, as it is a key medical ethical concept affecting abortion, human subjects protection in research, end-of-life care, futile and palliative care, and artificial respiration.

## MEDICAL HISTORY IN THE DEVELOPMENT OF CRITICAL AND ANALYTICAL THINKING

In my dual roles in premedical and medical education in the State of Qatar, one consistent observation has been that over the course of their medical training students’ cognitive tool kit, the rational strategies they employ for solving problems, slowly diminishes from an expansive set of skills during the premedical years to a more limited set of analytical tools, namely differential diagnosis, and variants of inductive and deductive reasoning, including the hypothetico-deductive method. Tools such as Bayesian networks, statistical inference, and the scientific method are the best means of establishing correct diagnosis, etiology, epidemiology, and evidence-based therapeutics, among other issues, but can prove inadequate when applied to complex multi-factorial phenomena such as human behavior, hospital ecosystems, medical ethics scenarios, and moral reasoning. The traditional humanities, on the other hand, have developed a range of strategies to address complex questions holistically within the categories of medical experience which cannot be broken down into smaller parts by analysis; for example self-reflective practice, intuitional knowledge and emotional intelligences (empathy), language-based tools (analogy, symbolic and metaphorical reasoning), and narrative approaches such as the use of historical exempla, parables, homilies, and proverbs to sharpen ethical inquiry, to provide just a few brief examples.

Within the popular paradigm of self-reflective practice, medical students and practitioners of all religious creeds and cultural backgrounds should be aware of their biases, and how they situate themselves culturally and religiously with respect to their patients – this is the essence of culturally competent care. The Muslim majority nations are extremely diverse with respect to history, culture, and language, even though they share a common religion. Core cultural rituals, customs, and world views are remarkedly resilient in Islamic societies, and the anti-westernization and anti-colonial movements that swept the Muslim world in the 1960s and which culminated in the wave of traditionalism and conservatism following the Iranian Revolution in 1979 have reinforced traditional cultural patterns. Therefore, historical reflection will necessarily form a central part of any Muslim healthcare worker’s self-reflective practice, for example, harmonizing traditional Quranic learning and ways of life with the forces of modernity, globalization, and the scientific doctrines such as the theory of evolution that contradict religious narratives.

One consequence of Saudi Arabia’s international mosque- and madrasah (kuttab)-building soft power diplomacy strategy was the widespread distribution and formal teaching of ultra-conservative and literalist Wahhabist texts such as *Kitab al-Tawhid* (*Book of Oneness [of God]*). The subtext of Ibn ‘Abd al-Wahhab’s concept of *Tawhid* was that there is only one interpretation of Islam, leading to a common belief among many adherents that Islam is monolithic. This was recognized as an extremist view even by his eighteenth-century contemporaries, and the resurgence of Ibn ‘Abd al-Wahhab’s works has been linked to modern *jihadist* ideologies. Due to the socioeconomic, cultural, and religious complexity of the patient populations in Muslim majority nations, therefore, the author advocates the formal teaching in medical schools of Islamic diversity, including the history of the peoples of the pre-Islamic Middle East, and such unpleasant historical topics as Arab colonization of North African and Persian cultures and the East African slave trade. Most of the current ethnic and political conflicts of the Middle East can be understood with just a cursory review of history.

A short course in Islamic epistemologies would be another welcome addition to Middle East medical school curricula in which historical and modern modes of Islamic thought could be introduced and compared analytically with the scope, methods, and aims of common medical modes of rational inquiry such as the scientific method, clinical decision-making, and medical ethical reasoning, with an emphasis on using the best epistemological tool in the right cultural and clinical context. Two brief examples of Islamic epistemological processes (ways of knowing) would include: first, the legal reasoning of Shariah law as pertaining to ethics determinations, including *qiyas*, consent of scholars, and public benefit (*maslaha*); and secondly, the centering of all knowledge in the Quran, with modern knowledge believed to derive analogically, symbolically, or metaphorically from the Quran’s eternal and comprehensive framework. Serious attempts were made in the 1980s and 1990s by such scholars as Seyyed Hossein Nasr and Ismail Al-Faruqi to Islamicize both science and knowledge.^13^ An often-cited example of the Islamicization of knowledge includes embryologist Keith L. Moore’s claim and subsequent textbook written with Abdul Majid al-Zindani that modern embryology is described accurately in the Quran.^14^ Moore and al-Zindani, however, appear to have been unaware of previous similar religious texts, the Alexandrian anatomists, or the embryological work of Aristotle which are likely sources of the Quranic passages.

## PARADIGM SHIFTS: UNDERSTANDING HISTORICAL DISEASE MODELS AND ILLNESS BELIEFS

In multicultural patient populations, patients often hold widely divergent beliefs about why they become sick. Responses range from views that disease is caused by external forces such as supernatural beings (and malevolent forces in Islam such as *djinn*, *al ayn*, or *hasad*) or biological agents (microbes or germs) to attitudes that disease is the result of internal imbalance (the hot/cold paradigm of Hispanic, Indian, and Chinese medicine, and the humoral model of classical Greek cultures). All of these illness beliefs (disease models) originate in deeply held historical modes of thought intimately related to culture, and are often firmly embedded in linguistic metaphors. Patients’ beliefs about illness can affect their acceptance of a treatment plan, their use of alternative medicine, or may even result in avoidance of evidence-based medicine.^15^

While the majority of Muslims today accept western evidence-based medicine and receive their medical care in hospitals, remnants of earlier medical philosophies remain influential. For example, the still well-regarded *tibb al-nabi* or Prophetic Medicine (discussed below) does not distinguish between physiological illness and illness arising from religious disbelief, doubt, and failure to follow Muslim rituals. All foods mentioned in the Quran (except alcohol), for example, talbeenah, olives, pomegranates, figs, dates, honey, are believed by Muslims to be medicinal in nature. Muslims do not automatically seek psychiatric and psychological services when experiencing abnormal behavior due to the general belief that possession by spirits is the cause of mental illness. These persistent illness beliefs obviously affect patients’ relationships with health systems and practitioners in numerous ways.

Fatalism and resignation represent the most common Muslim stances toward illness and misfortune due to the dogma that Allah propels all of creation and sanctions all suffering. According to the *Quran*, “no affliction comes about but by Allah’s permission” (64:11). The Quran also promises that “so verily, with the hardship, there is relief” (94:5). To counter surrender to illness, Hanbali jurist Ibn Qayyim Al-Jawziyya (1292–1350) in his compilation *Prophetic Medicine* espoused *ijtihad* or “struggle” and “effort” to regain wellness.^16^

Another widespread belief in Islam is that suffering during illness is a process of purification, and brings about expiation of sins and benefits in paradise: “No Muslim is afflicted with any harm …but that Allah expiates his sins because of that” (Sahih Bukhari, 70.551). According to Dif,

Muslims know that they do not have the power that permits them to master certain circumstances of their existence, in particular that which touches their health….they understand that Allah is the master of everything, of his destiny specifically. Every human being must live by passing many trials, which are however tests imposed by Allah to distinguish the sincere believers from the hypocrites.^17^

The belief that one’s being belongs entirely to God is consistent and widespread in Islam and informs key medical ethical concepts related to the sense of autonomy, fatalism, and the strong prohibition against suicide.^18^ In their roles as comforters and counsellors, a basic knowledge of the traditional role of the Quran and hadith would be helpful for physicians to guide Muslim patients towards an understanding of disease within standard Islamic orthodoxy. As an example, the author’s Muslim medical colleagues often remind patients who refuse treatment (believing that their illness is fated by God) of the following hadith: “The Prophet (ﷺ) said, ‘There is no disease that Allah has created, except that He also has created its treatment’” (Al-Bukhari, 76.1).^19^

In summary, the study of the history of medicine introduces the key concept of paradigm shifts and how dominant medical philosophies (including spiritual forces, humoralism, germ theory, genetic medicine, personalized medicine, and others) have risen and fallen. Through historical appreciation of different philosophies of medicine, practitioners then become more aware of the economic, social, and cultural dimensions of illness. The big-picture thinking involved in tracing historical processes in medical history research thus encourages practitioners to take a wider view of their own practices which takes into account their ethical, practical, social, and economic roles in society. History, as a reflection of culture, also provides insights into the patient perspective on disease and the profession of medicine itself.

## UNDERSTANDING CULTURE AND RELIGION: CLINICAL CULTURAL COMPETENCY

The works of Vanessa Gamble and Joel Howell provide the most compelling arguments for requiring historical competency in physicians to aid them in delivering culturally sensitive healthcare. Gamble documented fears expressed in the African American popular press in the 1980s and 1990s during the AIDS crisis that medical doctors were either experimenting on Black patients or that the AIDS virus had been purposefully engineered to unleash genocide on Blacks in America.^20^ These fears can be traced to the revelations of the Tuskegee syphilis experiment in 1972 and to earlier nineteenth-century experiments on enslaved Africans by White doctors such as James Marion Sims.^21^ Howell in 2005 confirmed Gamble’s findings with a poignant clinical anecdote that clearly demonstrated the impact of historically-based distrust on patient outcomes. He demonstrated that avoidance of medical care due to racial factors resulted in lower patient adherence, compliance, and satisfaction.^22^ Likewise in Muslim-majority countries, patient beliefs and behaviors and their relationships with their providers can often be clearly linked to the historical factors that formed their belief systems. A formal knowledge of these historical and cultural traditions can provide diagnostic clues and aid in holistic treatment through the development of trust relationships, information sharing, and shared decision-making.

## TRADITIONAL MEDICINE AS A CONTINUING HEALTH PRACTICE IN MUSLIM MAJORITY COUNTRIES

Traditional folk medical practice is prevalent throughout the Middle East and North Africa (MENA) region particularly in non-urbanized areas. Traditional practices historically originate from four sources: 1) pre-Islamic oral traditions, “Bedouin medicine;” 2) *tibb al-nabi* or medicine of the Prophet, which comprises health information contained in the Quran, hadith, and sunnah of the Prophet Mohammed (SAW); 3) remnants of Akkadian, Egyptian, and Persian medicine and; 4) remnants of Greek humoralism, *Unani Tibb*. According to Al-Kindi, as of 2011 traditional medicine is still widely used in Oman despite the widespread availability of modern medicine.^23^Al Saeedi additionally reported in 2003 that 30.1% of 1039 diabetic patients surveyed in Saudi Arabia were using traditional herbal medicines such as black seed (Habbah soda, *Nigella sativa*), garlic (Thoum, *Allium sativum*), and Murrah (myrrh, *Commiphora spp.*).^24^

A historically-informed practitioner would know the origin and makeup of traditional medicines, since some of them are highly toxic or invasive while others are quite effective and can be recommended within the Traditional Medicine/Complimentary and Alternative Medicine (TM/CAM) model of the World Health Organization, especially for low-income patients. For example, the traditional herbal mixture Za’atar containing *Thymus vulgaris* is an effective and non-toxic mouth and throat antiseptic used for centuries in Arabian medicine (active ingredient thymol). Practitioners must also be informed about the historical dimensions of folk medicines as well and how they have developed, since many of them possess cultural and religious significance. Faith-based therapies such as Quranic recitation can be successfully suggested to patients as adjuvant therapy to modern provenly efficacious interventions. This training helps clinicians negotiate modern allopathic and traditional treatment strategies with patients and families.

Effective traditional topical and internal antibiotics, anti-diarrheals, antipyretics, and other agents have been identified through randomized controlled trials (RCTs). However, the medical case study literature of traditional treatments in the MENA region contains numerous reports of eschars from cauterization (*wasim*, *kaii*), improper alignment of fractures from *tajbir* (bonesetting), infection or scarring from bloodletting and cupping (*hijama*), or heavy metal poisoning (Hg, Sb, Pb) arising from such traditional preparations as *kohl* (antibiotic *eyepaints*; active ingredients, *ithmed* = antimony and galena = lead sulphide) and *bint al-dhahab* (daughter of gold, containing PbO, antimony and cadmium). Lead encephalopathy in children has been reported in regions that use *bint al-dhahab* or kohls on children, and its importation is now banned in many Middle Eastern countries.^25^

## MENTAL ILLNESS IN ISLAM, AND THE DECLINE OF MUSLIM INTELLECTUAL TRADITIONS

Psychiatric and psychological training and practice are areas in which a knowledge of the history of medicine is essential for Middle Eastern practitioners. Western theories of mind, cognition, epistemology and behaviorism are not widely accepted outside of scientific and medical communities in Muslim majority countries, and the causes of mental aberration are popularly ascribed to spiritual and metaphysical forces. In a 2019 study from Saudi Arabia, “82.2% of the participants reported using one or more types of CAM therapies within the past year to address mental illness” with the number one therapy being Quranic recitation (including exorcism).^26^

In a cross-sectional study of 2,514 subjects in Qatar in 2010, Bener and Ghulom reported that 43.5% of men and 34.5% of women believed that mental illness was caused by evil spirits, such as *djinn* (genies).^27^ It is obligatory (*fard*) for Muslims to believe in *djinn* since they are unambiguously described in the Quran, and in most Muslim-majority countries *djinn* possession is widely believed to be the main cause of mental illness, often cured by exorcism rituals (*ruqya*).^28^

Ameen lists the following mental conditions caused by Shaytan (Satan) spirits or *djinn*: “intense fear…psychological and nervous diseases, insanity, depression, anxiety, tension, epilepsy, Waswaas (whispers from the Shaytaan), personality disorders…hallucinations.”^29^ In Saudi Arabia, Al-Habeeb reported that traditional Islamic medicine ascribed serious mental disorders such as psychosis, aphonia, or delusions to either *as-sihr* (sorcery) or *djinn* possession.^30^ Medieval Muslim physicians also wrote about madness by fusing the materialist tradition of Galenic and pseudo-Aristotelian texts with the sparse discussions of personhood (*nafs*) and spirits (*ruh*) scattered throughout the Quran. Early Islamic physicians such as Ibn Sina (980–1037) and Al-Razi (854–925) drew heavily on Aristotle to aid them in understanding the soul and ‘Ali ibn al’Abbas al-Majusi (Latin: Haly Abbas) in his *Kitab al-Maliki* described many neurological diseases such as hemiplegia, amnesia, and epilepsy in a purely clinical fashion. Al-Majusi’s book was widely reprinted in the Latin translation by Constantinus Africanus called the *Liber pantegni*; thus, the Greek psychological heritage was expanded upon by the clinical observational work of Islamic physicians, who were foundational to early psychological theory in Europe.

Clearly, both Western and Islamic psychological theories therefore share some common philosophical roots and a shared textual traditional. This historical fact, when discussed with Muslim patients, may increase acceptance of western psychiatry. The individual practitioner in the Middle East will need to decide how to negotiate the difficult terrain of differing beliefs on disease origins with patients and families, since pharmacological and psychiatric approaches to mental illness developed in Western countries are not acceptable to all Muslim patients who are unfamiliar with modern psychology’s common origins; dismissing outright supernatural origins of mental illness risks offending patients and challenging deeply held belief systems, resulting in non-adherence and non-compliance.

## ISLAMIC MEDICAL ETHICS

The emerging discipline of Islamic medical ethics, initiated during the 1980s to the 1990s following medical innovations such as organ transplantation, IVF, and artificial respiration, should be taught in medical schools in all Muslim-majority countries in tandem with a basic historical grounding in *fiqh* (law), since many laws and regulations governing medical practice today in these countries can be directly traced to late medieval *fatawa* (opinions) of the recognized schools of law (*madhhabs*): the four Sunni schools (Maliki, Hanafi, Shafi’i, Hanbali), the two main Shia schools (Zaidi, Ja’fari), the Ibadi madhhab (Oman), as well as numerous other historical schools. Rulings of these schools must be interpreted within historical context, since each school deliberates in a different manner, and is dominant in a particular region of the Muslim world, therefore reflecting local customary law (*‘urf*). In a few countries, shariah law courts constitute the primary legislative system, while in others they rule only on family matters, and in some cases they compete for authority with civil courts. The Hanbali school operates over most of the Arabian peninsula and is aligned with Salafism, thus its rulings are strict and follow textual authority closely. The more liberal Shafi’i school can be found across Africa and Asia, specifically the Horn of Africa and Indonesia, and its rulings are often quite different from other Sunni schools as the local populations there are not Arabs.

Why should these legal technicalities concern the Muslim medical student or practitioner? Although Imams, Muftis, sheikhs and Mullahs are respected and revered for their wisdom, it is not obligatory to follow their counsel, since Sunni Islam has no formal clerical structures nor can any Muslim claim infallibility. Thus individual Muslim practitioners and patients must make their own medical ethical decisions within their particular Islamic religious belief system in novel situations if there are no extant rulings. Who can speak authoritatively on medical ethics in Islam is a contentious and evolving question among schools of fiqh, governments, scientists, and physicians but the debates are clearly anchored in medieval history. Thus, to productively join these debates, students and physicians will need some grounding in the historical context and historical development of Islamic medical ethics.

Medical ethics itself can properly be said to have originated in the pre-Islamic Babylonian empire with the Code of Hammurabi (c. 1754 BCE) which describes medical liability of physicians for killing patients. Both the Aristotelian and Hippocratic ethical traditions can be traced within medieval Islam, and the ninth-century author Al-Ruhawi produced a lengthy treatise on ethics entitled *Adab al-Tabib* (*Ethics of a Physician*), a mixture of deontological and virtue ethics.^31^ Although there are gaps in the textual history, Islam therefore has a long tradition of medical ethics that can be productively consulted in forming modern opinions.

Islamic medical ethics is primarily deontological, stemming from duties outlined in a hierarchy of texts consisting of the Quran (Allah’s revealed truth to Prophet Mohammed [SAW]), the Sunnah (acts of the Prophet [SAW]), and Ahadith (collected sayings of the Prophet [SAW] attributed to him by other speakers). As I have explained elsewhere, “if these texts cannot provide unequivocal answers, jurists turn to the consent of scholars (*ijma’*), analogies (*qiyas*), and then finally invoking the principle of *maslaha* (public benefit) if necessary.”^32^ Reasoning from *maslaha* is a form of consequentialist thinking most akin to Utilitarianism and is becoming a more widely used deliberative technique in Islamic bioethics as a result of highly novel developments in biomedicine, such as in vitro fertilization and artificial reproductive technologies, stem cells, and robotics which have no analogues in sacred scriptures. Recently, biomedical researchers have been meeting with Muslim jurists at special international congresses to create policies and issue opinions. Thus modern-day Muslims in healthcare professions will need to acquire some basic knowledge of the historical madhhabs and their reasoning processes in order to participate productively in emerging medical ethical debates which affect Muslim patient populations and research subjects.

## ISLAMIC MEDICAL ETHICS AS A DISTINCT DISCIPLINE: CULTURAL DIFFERENCES IN DISCLOSURE AND PATIENT AUTONOMY

Full medical information disclosure has become the norm in western societies due to the emphasis on patient autonomy and the patients’ rights movement. Historically, however, disclosure of information to patients and patient autonomy manifests quite differently in traditional Muslim families who follow the *wali* (guardianship) or *mahram* system. Families always expect to be part of the decision-making process of their individual family members, and families commonly ask physicians not to reveal fatal illnesses to a patient, particularly to the very young and very old. According to Al-Bahri, “in certain Middle Eastern, North African and South Asian societies, the family has final authority in [treatment decision-making], as often the patient is inseparably linked to the family as a whole.”^33^ In a survey of 200 Bahraini physicians in 2019, over 85% indicated that they would make an exception to full disclosure of information to the patient, particularly if requested by the family, and in another survey of 164 Jordanian physicians, a surprising 23% indicated that they “usually withheld the diagnosis of serious illness from patients.”^34^ The author has personally encountered numerous instances in the Gulf region health systems in which historical medical services provision based on traditional culture (both medical and social patriarchalism) has clashed with a growing patients’ rights movement and the growing autonomy of young people, due to increasing modernization and westernization and the decline of large extended family structures. Awareness of, and perhaps even formal training in, historical modes of provider-patient relations in Islamic societies and shared decision-making processes would be helpful for providers-in-training.

## PROFESSIONAL IDENTITY DEVELOPMENT: NARRATIVE MEDICINE, ROLE MODELLING, AND THE PROFESSIONAL WORKPLACE

Although there is the risk of perpetuating Thomas Carlyle’s “Great Man Theory of History,” a more gender and racially inclusive list of medical and scientific heroes and heroines from the past who embodied both the moral behaviors and high level of technical proficiency encouraged in modern medicine (for example, selflessness, benevolence, intellectual rigor, observational precision, and competence) may prove valuable in inspiring and instructing physicians and students at all stages of their careers.^35^ The comprehensive assessment known as the “Flexner II Report,” detailing the state of medical education in the US and issued by the Carnegie Foundation 100 years after Abraham Flexner’s foundational report in 1910, concluded that greater attention needs to be paid to physician identity formation in medical education.^36^ We ask healthcare professionals to be sensitive to their patients’ race, religion, gender, and culture. Why not ask doctors to be culturally literate about their own profession and to examine their own subject positions? A thorough understanding of the self, including one’s professional identity, has long been viewed as a pathway to knowledge, for example in the tradition of the Oracle of Delphi’s “know thyself.” Physician-historian Jeffrey Baker recounts how, in his view, historical research into the fields of pediatrics and gynecology “transformed my self-identity….I returned from my historical journey with a different understanding of my professional relationship to other health care workers.”^37^ Doctors’ stories, such as Atul Gawande’s *Complications* (2002) or Jerome Groopman’s *How Doctors Think* (2007), are helpful in modelling professional behaviors and ethics for physicians-in-training, and Muslim doctors’ narratives from the late medieval to modern period are equally valuable.

Ibn Sina (Avicenna) is known to most Muslim school children: he wrote over one million words on medicine in the *Qanun fi al-Tibb* (the equivalent size of a modern multi-authored internal medicine textbook) and claimed to have mastered the medical knowledge of his time by age eighteen. He wrote on all the topics comprising the medieval quadrivium and trivium and once joked “medicine is not one of the difficult sciences, and therefore I excelled in it in a very short time.”^38^ In addition, his synthetic method stands as a prime example for modern Muslim medical practitioners and students that philosophy, empiricism, and medical science are not incompatible with Islam. Therefore he represents a valuable counterweight to the postcolonial nationalist discourses that western science is inimical to the Islamic world view and corrosive to Islamic values. Abu Bakr Muḥammad b. Zakariyāʾ Rāzi (Al-Rāzi) has been hailed, in the same way as Hippocrates in *Epidemics I, III* and *On the Surgery*, as a timeless model for close clinical observation. He was the first physician to differentiate measles from smallpox, in *al-Jadari wa’l-ḥaṣba (On Smallpox and Measles),* through careful observation.^39^ Although Al-Razi’s *Kitab al-Mansuri* and *Kitāb al-Ḥāwī fī al-ṭibb* are based on several recognizable sources (Aristotle, Aetius of Amida, Paulus Aegineta, Oribasius, Galen, Hippocrates), *On Smallpox and Measles* appears to be largely original, an important and inspirational fact for Muslims since novel scientific knowledge production in Muslim-majority countries today lags well below western countries according to all standard metrics.^40^ He also stands as a reminder against dogmatism in medicine when he argued in the *Shukuk al-jalinus (Doubts about Galen)* that physicians should not rely solely on previous authority but rather question everything.

In the modern period, woman physician Nawal El-Saadawi has been inspirational to Muslim doctors in the region for decades due to her advocacy for women in medicine, human rights in Islam, and efforts against female genital mutilation. Born in 1931 in rural Egypt, she has written several memoirs, including a 3-volume autobiography and an account of her time in prison. The historical insights from her 65-year career in medicine are particularly valuable since they chronicle dramatic shifts in women’s professional roles in medicine in the Middle East circa 1955 to 2021.^41^ From reading her works, younger women physicians who now benefit from greater opportunities ushered in by the second-wave feminist thinking that she inspired will understand the obstacles that their older women colleagues faced. Readers of El-Saadawi will gain additional insights into older patients who experienced medical treatment and provider-patient relationships in a very different manner in the past (for example, extreme medical patriarchalism). El-Saadawi’s books on violence against women in Islam could also initiate research into the relationship of the history of gender violence and social hierarchies to bullying, harassment, and undermining in Middle East healthcare systems (an almost entirely neglected topic at the present time), which have now been recognized and partially addressed in developed nations through an examination of what has been characterized as the “hidden curriculum” in education.

Thus literature on pioneers and exemplary clinicians in Middle Eastern medicine could fill a gap for modelling desired behaviors in medicine where comprehensive physician competency frameworks are currently lacking. For instance, one of the few competency regimes in the Middle East, a medical professionalism framework of nine core domains, was only recently developed in 2016 in the United Arab Emirates (UAE) by consensus of a select group of Muslim medical authorities. Similar official frameworks are exceedingly rare in the Middle East as it is generally assumed that a good Muslim will also be a good and ethical physician. The UAE framework resembles western antecedents such as the UK General Medical Council’s *Good Medical Practice* and Canada’s CanMEDS in most respects except for item number one, “Commitment to *Ihsan* and adherence to ethical practice” which is described as the “Physician’s pursuit of professional practice should derive from an internal sense of duty to pursue *Ihsan*, a sense of social responsibility from the belief that he or she is accountable to a higher being.”^42^ The term *ihsan* means to do beautiful (moral) deeds as if God were watching the believer and the inclusion of this item underscores again how Muslim societies frame all human activity including the profession of medicine within the scope of religious practice. Due to the highly multicultural makeup of the UAE (Emiratis account for only 12% of the total population, and are primarily Sunni Muslims), the new guidelines are problematic for atheists and non-theists.

## WORKPLACE ORGANIZATION

Islam has developed several unique historical models and methods of care that have affected modern institutions in the Middle East and which can be revisited in imaging new ways of services provision. The Bimaristan (Persian: “place of the sick”) provided publicly-funded health services and physicians financed directly by the Caliph often cared for the poor and the incurably insane. Notable was the use of aesthetics to aid in healing (based on Arabic-Galenic theories of the impact of environment on disease); in other words, Bimaristans provided a pleasant environment including gardens, open courtyards, and fountains which were used for hydrotherapy to calm patients, in addition to music therapy. Also, inspirational Quranic verses involving illness were often inscribed in the stone-work illustrating the belief that the Quran itself and recitation of its text provides healing powers.^43^ As part of the Islamic architecture revival of the past decade, Qatar’s multi-billion dollar Sidra Women’s and Children’s hospital in Doha, Qatar incorporated many traditional elements of the bimaristan, such as healing gardens and water.

Many hospitals historically and today throughout the Muslim world are endowed through the system of *waqf*, or perpetual charitable endowment. This practice not only serves to redistribute wealth and prevent the intergenerational accumulation of resources among Muslim families, but also reminds the community of the duty to provide for the sick according to the fundamental Islamic pillar of Zakat or charity. In conclusion, health policy or service provision change can only occur if one is willing to imagine other ways of doing things, and the past constitutes a reference manual of different institutional structures, care models, and professional behaviors.

## MEDICAL HISTORY IN THE MIDDLE EAST IN ACTION: SAMPLE ACTIVITY TO HARNESS HISTORY FOR REFLECTION IN MEDICAL ETHICS

In 2006, I developed the following activity on medical oaths, which I have presented to clinicians in ACCME training workshops and medical and premedical students in humanities classes to promote reflection on medical ethics and professionalism. The project is generally preceded by a module on Islamic medical ethics and/or the history of Islamic medicine.

## OATH PROJECT

Participants first study a packet of historical medical oaths (original Hippocratic Oath–Edelstein translation, Maxim of Kung Hsin, WMA 1948 Declaration of Geneva, Islamic Medical Oath of IMANA and IOMS, Oath of Asaph, Oath of Maimonides, etc.). After one to two-hour lecture sessions which situate each oath historically, participants engage in small group discussion. Since a modified form of the Hippocratic Oath is the most popular oath administered at US medical schools (including the author’s own medical school), the majority of one entire session is devoted to Hippocrates, the Hippocratic ethical tradition in the context of Islamic *Adab*, and a comparison of the original oath with modern and Islamic versions.^44^ Some guiding discussion questions posed are: do universal ethical principles in medicine exist? Should each religion or ethnic group have their own oath? Are medical oaths practically useful or simply ceremonial, and should they be enforced? Sometimes Muslim participants express unease at the concept of a secular oath, believing that oaths can only be sworn to Allah.

Participants then write their own oath summarizing in bullet point form or aphoristic style their views on the appropriate rules and guidelines of conduct of a healthcare worker (or if they do not believe in the value or practicality of oaths to guide conduct, they can prepare arguments against the use of oaths). A debriefing and group discussion is then led by the instructor. The activity collates past wisdom on moral behavior in medicine from previous medical oaths, and allows for reflection on the core principles of physician ethical conduct as well as the purposes and practicality of professional oaths.

## WHAT WOULD A MIDDLE EAST MEDICAL SCHOOL CURRICULA FOR THE HISTORY OF MEDICINE LOOK LIKE?

The preceding discussion has hopefully established that history and historical precedent continue as a living tradition in all facets of Islamic life, including medical training and practice. I therefore recommend that medical schools in Muslim-majority nations formally teach Islamic medical history in conjunction with medical ethics and cultural competency, and that universities develop related specialized courses taught by relevant experts on Islamic law, history, ethics, and theology for Continuing Medical Education. Realistically, a three to six month medical school course would integrate the following humanistic topics in the suggested syllabus below:

**Table:** 

Islamic Medicine: History and Applications	
TOPIC	RATIONALE AND LEARNING GOALS
Islamic Medical Ethics	Trace the development of decision-making in medieval thought, *fiqh* councils, internet *fatawa* (opinion) sites, and Muslim medical associations. Discuss modern ethical issues in the context of historical precedent. Analyze deliberative processes and landmark historical rulings and principles. Texts: al-Ruhawi’s *Adab al-Tabib*, “Practical Ethics of the Doctor,” and modern overviews such as Sachedina and Atighetchi.^45^
Cultural Competency	Provide an overview of the history of pre-Islamic and Islamic cultures leading to current cultural diversity in the Muslim world. Discuss differences in Islamic sects and how culture and religion affect medical services provision and the provider-patient relationship. Texts: Nasr’s *Islam: Religion, History, and Civilization* or Armstrong’s *Islam: A Short History*.^46^
Islamic Ways of Knowing (Epistemology)	Explore historical means of understanding the world through scripture, debate, logic, experiment, etc. with reference to traditional knowledge systems in Islam. Texts: Excerpts from the works of Ibn Sina, Al-Ghazaly, Ibn Rushd, Yusuf Al-Qaradawi, and historian of Islamic philosophy Peter Adamson.
History of Science and Medicine in Islam	Demonstrate common medical traditions in Eastern and Western societies, and discuss core principles that led to medical progress (close observation, professional frameworks, desire for knowledge, *lingua francas*, scientific networks and research and translation centers – *bayt al hikmah*, etc). Texts: chapters from standard reference works; currently there is no accurate, brief, and comprehensive non-specialist overview of the topic with the exception of Khan and Ullmann.^47^
Medical Luminaries in Islam	To instill pride in Muslim medical students and serve as guides in ethics and practice. Texts: entries from biographical dictionaries and encyclopedias.
Traditional and Folk Medicine	Learn about continuing folk medical practices and their relationship to history and religion, with emphasis on mental illness. Understand the actual practices and the ingredients in current therapeutic substances and their potential benefits, harms, and contraindications. Discuss strategies to negotiate common Muslim patient perceptions that mental illness is supernatural in origin. Texts: article packet.

## CONCLUSION

In conclusion, I suggest that healthcare practitioners in Muslim majority countries educate themselves or seek continuing professional development training at a university on the historical dimensions of medicine in the region in order to:

1. trace the history of Muslim thought (philosophy and theology), with special reference to how medical ethics decisions are currently decided in various madhhabs of shariah law to inform their clinical and research practice,
2. gain knowledge of the history of the Islamic caliphates and the cultural diversity of the Middle East arising from indigenous cultures to better empathize and communicate with the range of peoples living in the region (cultural competency),
3. understand traditional folk medical practices, including religious healing. Physicians will hopefully over time build a detailed knowledge base about toxic ingredients in traditional compounded and herbal medicines, as well as dangerous pharmacological synergies and contraindications. Physicians will also be able to develop strategies to talk to patients about TM/CAM usage, and traditional beliefs that clash with western evidence-based medicine,
4. develop an interest in medical luminaries from the history of Islamic medicine – Avicenna, Albucasis, Rhazes, and others – to inspire them, and their students, patients and colleagues,
5. be open to traditional attitudes towards mental illness as demon possession, and offer resistant patients options for pharmacological interventions, group and expressive therapies, and Cognitive Behavioral Therapy (CBT) as adjuvant therapy to Quranic recitation and rituals and charms instead of dismissing culturally-relevant cures and alienating patients.

## Footnotes

1 Islam is not monolithic in any sense, with a range of sects from Sunnis, Shias, Ibadhis, Deobandis, Salafis, Sufis, Ismā’īlīs, etc. as well as numerous historical Kharijite sects. A Muslim is defined herein as anyone subscribing to the five *arkān ad-dīn* (­pillars of faith: Shahada, Salat, Zakat, Sawm, and Hajj). Likewise the term Middle East is an inexact and ethnocentric geopolitical category, and herein designates the Arab, Persian, Tamazight, and Turkic cultures that were influenced by Islam.

2 The literature on the inclusion of medical history in medical education has been comprehensively summarized in David S. Jones, Jeremy A. Greene, Jacalyn Duffin, John Harley Warner, “Making the Case for History in Medical Education,” *Journal of the History of Medicine and Allied Sciences* 70:4 (2015), 623–652. However, there is almost no empirical longitudinal data tracing the impact of medical history training on subsequent clinical practice.

3 Specific student learning outcomes for WCM-Q have been reported elsewhere: Alan S. Weber, Utsav Nandi and Sabrina Alam, eds., *An Exploration of Literature and Medicine at Weill Cornell Medical College* (Doha: Doha Modern Printing, 2007); Alan S. Weber, “Medicine and Literature Courses for Arabic Speakers,” *Romanian Journal of English Studies* 7(2010): 427-44; Alan S. Weber, “Narrative Medicine in the Training of International Medical Students,” in *Pedagogical Education: Contemporary Problems, Modern Concepts, Theories and Practices*, ed. I.I. Sokolova (St. Petersburg: Russian Academy of Education, 2009), 162-66; Alan S. Weber, R.X. Sharkey, M.A. Rishel, Ian Miller, K. Golkowska, eds., *Qira’at: Readings from the Students of the Weill Cornell Medical College in Qatar*, Volume 4 (Doha: Dar Al Kutub Publishers, 2010).

4 Film: *1001 Inventions and the Library of Secrets*, available at https://www.1001inventions.com/films-and-videos/; Salim T. S. al-Hassani, ed., *1001 Inventions: The Enduring Legacy of Muslim Civilization*, 3rd edition (Washington, DC: National Geographic Society, 2012); for a detailed list of misrepresentations and historical errors, see Sonja Brentjes, “1001 Inventions… [Review],” *Aestimatio* 10 (2013), 119–153.

5 Rabie E. Abdel-Halim, Khaled M. AlKattan, “Introducing Medical Humanities in the Medical Curriculum in Saudi Arabia: A Pedagogical Experiment,” *Urology Annals* 4:2 (2012), 73-79, 76.

6 On remnants of tribalism in the Muslim world, see Miriam Cooke, *Tribal Modern* (Berkeley: University of California Press, 2014).

7 Stephen Frederic Dale, “Ibn Khaldun: The Last Greek and the First Annaliste Historian,” *International Journal of Middle East Studies* 38 (2006), 431-51.

8 Sidney Ross, “Scientist: The Story of a Word,” *Annals of Science* 18:2 (1962), 65-85, 71.

9 Ibn Khaldun, *The Muqaddimah: An Introduction to History*, trans. Franz Rosenthal, volumes 1-3 (London: Routledge and Kegan Paul, 1958).

10 E. Savage-Smith, S. Swain, and G.J. van Gelder, eds., *A Literary History of Medicine* (Leiden: Brill, 2020).

11 A. S. Weber, “Islamic Scholars on Ensoulment: An Historical and Contemporary Genesis,” in *Biruni Commemoration Volume*, ed. Abu Musa Mohammad Arif Billah (Dhaka: Abu Rayhan Biruni Foundation, 2018), 5.

12 Mansooreh Saniei and Raymond De Vries, “Embryonic Stem Cell Research in Iran: Status and Ethics” *Indian Journal of Medical Ethics* 5:4 (2008), n.p.; Ziaei, Saeideh and Mohsen Farokhi, “The Ethical Challenge of Stem Cell Research and Tissue Transplantation,” *Eubios: Journal of Asian and International Bioethics* 14 (2004), 97-99, 97.

13 Ismail al-Faruqi, *Islamization of Knowledge* (Herndon, VA: International Institute of Islamic Thought, 1982); S. H. Nasr, *Knowledge and the Sacred* (Albany: State University of New York Press, 1989).

14 Keith L. Moore, Sheikh Abdul-Majeed A. Azzindani, *The Developing Human: Clinically Oriented Embryology with Islamic Additions* (Saudi Arabia: Abul Qasim Publishing House, 1983).

15 Ann Fadiman’s *The Spirit Catches You and You Fall Down: A Hmong Child, Her American Doctors, and the Collision of Two Cultures* (1997) is one of the classic texts about the clash of disease philosophies.

16 I.Q. Al-Jawziyya, *Medicine of the Prophet*, trans. P. Johnstone (London: Cambridge Texts Society, 1998).

17 Malika Dif, *La Maladie et la Mort selon L’islam: Rites et Comportement* (Paris: Editions Tawhid, 2003), 19-20.

18 Fazlur Rahman, *Health and Medicine in the Islamic Tradition: Change and Identity* (New York: Crossroad, 1987).

19 Sahih al-Buhkari, *Book 76*: Medicine, available at https://sunnah.com/bukhari/76#!

20 Vanessa Gamble, “Under the Shadow of Tuskegee: African Americans and Health Care,” *American Journal of Public Health* 87:11 (1997), 1773-1778.

21 Keith Wailoo, “Historical Aspects of Race and Medicine: The Case of J. Marion Sims,” *JAMA* 320:15 (2018), 1529–1530; KM Hoffman, S Trawalter, JR Axt, and MN Oliver, “Racial Bias in Pain Assessment and Treatment Recommendations, and False Beliefs about Biological Differences between Blacks and Whites,” *Proceedings of the National Academy of Sciences* 113:16 (2016), 4296-4301.

22 Joel Howell, “Trust and the Tuskegee Experiments,” in *Clio in the Clinic: History in Medical Practice*, ed. Jacalyn Duffin (Toronto: University of Toronto Press, 2005), 213-225.

23 R.M. Al-Kindi, M. Al-Mushrafi, M. Al-Rabani, I. Al-Zakwani, “Complementary and Alternative Medicine Use Among Adults with Diabetes in Muscat Region, Oman,” *Sultan Qaboos University Medical Journal* 11:1 (2011), 62-68, 63.

24 M. Al-Saeedi, A.G. Elzubier, A.A. Bahnassi, K.M. Al-Dawood, “Patterns of Belief and Use of Traditional Remedies by Diabetic Patients in Mecca, Saudi Arabia,” *Eastern Mediterranean Health Journal* 9:1/2 (2003), 1-5, 1.

25 D.A. Woolf, “Aetiology of Acute Lead Encephalopathy in Omani Infants,” *Journal of Tropical Pediatrics* 36:6 (1990), 328-30; M.A. Worley, P. Blackedge, P. O’Gorman “Lead Poisoning from Eye Cosmetic,” *British Medical Journal* 1:117 (1968); M.A. Worthing, H.H. Sutherland, K. Al-Riyami, “New Information on the Composition of Bint al Dhahab, a Mixed Lead Monoxide Used as a Traditional Medicine in Oman and the United Arab Emirates,” *Journal of Tropical Pediatrics* 41:4 (1995), 246-47.

26 M.H. Rajab, F. Jabri, B. Alghalyini, et al., “A Hospital-based Study of the Prevalence and Usage of Complementary and Alternative Medicine Among Saudi Psychiatric Patients,” *Cureus* 11:9 (2019), e5584.

27 A. Bener and S. Ghuloum, “Gender Differences in the Knowledge, Attitude and Practice Towards Mental Health Illness in a Rapidly Developing Arab Society,” *International Journal of Social Psychiatry* 57:5 (2011), 480-486. Similar figures are confirmed by S. Al-Adawi, A.S.S. Dorvlo, S.S. Al-Ismaily, D.A. Al-Ghafry, B.Z. Al-Noobi, A. Al-Salmi, D.T. Burke, M.K. Shah, H. Ghassany, S.P. Chand, “Perception of and Attitudes Towards Mental Illness in Oman,” *International Journal of Social Psychiatry* 48:4 (2002), 305-17.

28 See the exorcism manuals by Bin Juned, *Possessed!: Its Treatment and Prevention Detailing ‘Practices and Methods used to Repel the Evil of an Intrusive Spirit so as to Free Oneself from being Possessed by it*’ (Brunei: State Mufti’s Office, 2005); Wahid Abdulsalam Bali, *Fortifying Man Against Djinns and Satan and Lawfull Enchants from the Holy Coran and the Sunna* (Beirut: Dar Al-Kotob Al-ilmiyah, 2012).

29 Abu’l-Mundhir Khaleel ibn Ibraaheem Ameen, *The Jinn and Human Sickness: Remedies in the Light of the Qur’aan and Sunnah*, trans. Nasiruddin Al-Khattab, ed. Dr. Adbul Ahad (Alig.) (Riyadh: Darussalam, 2005), 52-53.

30 Tariq A Al-Habeeb, “A Pilot Study of Faith Healers’ Views on Evil Eye, Jinn Possession, and Magic in the Kingdom of Saudi Arabia,” *Journal of Family and Community Medicine* 10:3 (2003), 31–38, 35.

31 Martin Levey, “Medical Ethics of Medieval Islam with Special Reference to Al-Ruhawi’s ‘Practical Ethics of the Physician,’” *Transactions of the American Philosophical Society* 57:3 (1967), 1–100.

32 A.S. Weber, “Bioethical Reasoning in Islam,” *International Journal of Arts and Sci*ences 3:15 (2010), 607-617.

33 A. Al-Bahri, Al-Moundhri M, Al-Azri M, “The Role of Patients’ Families in Cancer Treatment Decision-Making: Perspectives among Eastern and Western families,” *Sultan Qaboos University Medical Journal,* 7 (2017), e383–5.

34 F. Naji, G. Hamadeh, S. Hlais, S. Adib, “Truth Disclosure to Cancer Patients: Shifting Attitudes and Practices of Lebanese Physicians,” *AJOB Empirical Bioethics* 6 (2015), 41–9; SM Borgan, JZ Amarin, AK Othman, HH Suradi, YZ Qwaider, “Truth Disclosure Practices of Physicians in Jordan,” *Journal of Bioethical Inquiry* 15 (2018), 81–7.

35 Thomas Carlyle, *On Heroes, Hero-Worship, and The Heroic in History* (London: James Fraser, 1841).

36 Molly Cooke, David M. Irby, Bridget C. O’Brien, Lee S. Shulman, *Educating Physicians: A Call for Reform of Medical School and Residency,* Jossey-Bass/Carnegie Foundation for the Advancement of Teaching, volume 16 (New York: John Wiley & Sons, 2010).

37 Jeffrey P. Baker, “Historical Adventures in the Newborn Nursery: Forgotten Stories and Syndromes,” in *Clio in the Clinic*, 107.

38 WE Gohlman, *The Life of Ibn Sina: A Critical Edition and Annotated Translation* (New York: State University of New York Press, 1974).

39 Max Meyerhof, “Thirty-Three Clinical Observations by Rhazes (Circa 900 A.D.),” *Isis* 23:2 (1935), 321-372; Richard Mead, *A Discourse of the Small-pox and Measles. To this is Subjoined The Commentary of Rhazes, a Most Celebrated Arabian Physician, on the Same Diseases* (London: A. Dodd; M. Nutt and E. Cooke; and E. Amey, 1747).

40 Oliver Kahl, *The Sanskrit, Syriac and Persian Sources in the Comprehensive Book of Rhazes* (Leiden: Brill, 2015).

41 Nawal El Saadawi, *Memoirs of a Woman Doctor* (San Francisco: City Lights Books, 2001); Nawal El Saadawi, *A Daughter of Isis: The Early Life of Nawal El Saadawi* (London: Zed Books, 2018); Nawal El Saadawi, *Walking through Fire: The Later Years of Nawal El Saadawi* (London: Zed Books, 2018); Nawal El Saadawi, *Memoirs from the Women’s Prison* (Berkeley: U. California Press, 1994). Due to her sympathetic treatment of prostitutes, El Saadawi’s books were banned at the author’s institution in Qatar in the early 2000s.

42 S. Abdel-Razig, H. Ibrahim, H. Alameri, H. Hamdy, K.A. Haleeqa, K.I. Qayed, L.O. Obaid, M. Al Fahim, M. Ezimokhai, N.D. Sulaiman, S. Fares, M.M. Al Darei, N.Q. Shahin, N.A. Al Shamsi, R.A. Alnooryani, and S.Z. Al Falahi, “Creating a Framework for Medical Professionalism: An Initial Consensus Statement From an Arab Nation,” *Journal of Graduate Medical Ed*ucation 8:2 (2016), 165–172.

43 Françoise Cloarec, *Bîmâristâns, lieux de folie et de sagesse* (L’Harmattan, Paris, 1998), 90.

44 AC Kao and KP Parsi, “Content Analyses of Oaths Administered at U.S. Medical Schools in 2000,” *Academic Medicine* 79:9 (2004), 882–7.

45 A. Sachedina, *Islamic Biomedical Ethics: Principles and Application* (Oxford: Oxford University Press, 2011); D. Atighetchi, *Islamic Bioethics: Problems and Perspectives*, International Library of Ethics, Law, and the New Medicine Book 31 (Geneva: Springer, 2006).

46 S.H. Nasr, *Islam: Religion, History, and Civilization* (New York: HarperOne, 2009); K. Armstrong, *Islam: A Short History*, revised edition (New York: Modern Library, 2002).

47 M Salim Khan, *An Introduction to Islamic Medicine* (Leicester, UK: Mohsin Health, 2018); Manfred Ullmann, *Islamic Medicine* (Edinburgh: University of Edinburgh Press, 1978).

